# Supplementary material for: Associations of the objective built environment along the route to school with children’s modes of commuting: A multilevel modelling analysis (the SLIC study)
Source: PLoS One. 2020 Apr 9;15(4):e0231478. doi: 10.1371/journal.pone.0231478 (PMC7145202; doi:10.1371/journal.pone.0231478)
Supplement: S2 Table — (DOCX) [file pone.0231478.s002.docx]

| ***Supplementary table 1*** | | |
| --- | --- | --- |
| ***Descriptive statistics for the sample of SLIC children included in the current study*** | | |
| **Variable** | **Number of SLIC children** | **Percentage (%)** |
| **Sex** |  |  |
| *Female* | 1 015 | 53.7 |
| *Male* | 874 | 46.3 |
| **Ethnicity** |  |  |
| *Black* | 490 | 25.9 |
| *South Asian* | 515 | 27.3 |
| *White/other* | 884 | 46.8 |
| **Age at test (years old)** |  |  |
| *5-6* | 464 | 24.6 |
| *7-8* | 720 | 38.1 |
| *9-11* | 705 | 37.3 |
| **ln(FMI) z-score**  *<5^th^ percentile*  *5^th^-85^th^ percentile*  *85^th^-95^th^ percentile*  *>95^th^ percentile* | 56  1 546  172  115 | 3.0  81.8  9.1  6.1 |
| **Family Affluence Scale** |  |  |
| *Low* | 165 | 8.7 |
| *Intermediate* | 1 208 | 63.9 |
| *High* | 396 | 21.0 |
| *Unknown* | 120 | 6.4 |
| **Free School Lunches** |  |  |
| *Yes* | 462 | 24.5 |
| *No* | 1 268 | 67.1 |
| *Unknown* | 159 | 8.4 |
| **Number of cars in the household** |  |  |
| *None* | 438 | 23.2 |
| *One* | 909 | 48.1 |
| *Two* | 434 | 23.0 |
| *Unknown* | 108 | 5.7 |
| **IMD Quintile** |  |  |
| *Least deprived: 1* | 177 | 9.4 |
| *2* | 372 | 19.7 |
| *3* | 356 | 18.8 |
| *4* | 375 | 19.9 |
| *Most deprived: 5* | 609 | 32.2 |
| ***School ID***  *School 1*  *School 2*  *School 3*  *School 4*  *School 5*  *School 6*  *School 7*  *School 8*  *School 9*  *School 10*  *School 11*  *School 12*  *School 13* | 105  63  130  97  280  85  209  150  81  249  181  139  120 | 5.6  3.3  6.9  5.1  14.8  4.5  11.1  7.9  4.3  13.2  9.6  7.4  6.3 |
