# Supplementary material for: Associations of the objective built environment along the route to school with children’s modes of commuting: A multilevel modelling analysis (the SLIC study)
Source: PLoS One. 2020 Apr 9;15(4):e0231478. doi: 10.1371/journal.pone.0231478 (PMC7145202; doi:10.1371/journal.pone.0231478)
Supplement: Attachment — Submitted filename: Response to Reviewers Bosch et al PLOS One.docx [file pone.0231478.s003.docx]

**Authors’ Response to Reviewers Manuscript *PLOS One***

Dear Madam, Sir,

Please find below our response to the reviewers’ comments on our manuscript entitled *“Associations of the objective built environment along the route to school with children’s modes of commuting: a multilevel modelling analysis (the SLIC study)”*. The authors thank the reviewers for their in-depth reading of the manuscript and their useful suggestions, which are responded to below on a point-by-point basis. We also updated our ‘Acknowledgments’ section, and added a copyright reference to figure captions. All changes made and the reviewed manuscript have been approved by all authors.

Best wishes,

Lander Bosch, corresponding author

### **Reviewer 1**

General Comments

1) *The manuscript is well written and structured in a scientific manner. It increases the knowledge of children’s modes of commuting. I would suggest the authors only to take into account instead of the large sample of the SLIC project (n=2171), the final sample of this study (n=1.889).*

We thank the reviewer for this observation, and took this forward in our manuscript. To facilitate the interpretation of our data and analyses, we have decided to only focus on the final sample of 1,889 children included in this study, instead of also referring to the entire SLIC cohort of 2,171 children. Reference to the latter has been removed from the ‘Abstract’, ‘Materials and Methods’ and ‘Results’ sections, more specifically on lines 35, 119, 259 and 264 in the revised manuscript.

Specific Comments

2) *Lines 116-117: The final sample from lines 257-258 should be described instead of the whole SLIC sample. The sex and the age distribution should be mentioned.*

In accordance with the reviewer’s general comment, we described only the final sample, both on lines 116-117 (line 119 in the reviewed manuscript), and lines 257-258 (line 264 in the revised manuscript), as well as on line 35 in the abstract. Moreover, we moved the sex and age distribution information from the ‘Results’ section on lines 334-340 to the ‘Materials and Methods’ section on lines 121-124 in the revised manuscript, as suggested by the reviewer.

### **Reviewer 2**

1) *Where there any differences in the characteristics of the children excluded and in those included in the analysis? Please add a comment/description.*

Due to the focus on the final sample, as suggested by Reviewer 1, references to the overall SLIC sample and excluded children are no longer included in the revised manuscript. For information, however, the children in the sample withheld for multilevel modelling did not differ significantly in terms of age, sex or ethnicity from the full SLIC sample. The excluded children, however, had a 7% higher proportion of males, were slightly older (by 0.2 years), and had a higher proportion of black students (49% versus 26% in the included sample). The latter is due to the fact that the lion’s share of missing data and consequentially excluded children stemmed from three schools with circa 75% black students.

2) *Should also the parents' PA/attitude be taken into account as possible confounders in the analyses? What about school scores? I think these could make a difference, too. Was such information available/asked? If not, perhaps they should be mentioned in the discussion as missing pieces of information.*

We agree with the reviewer that this information could have further added to insights provided by our study. However, as these data were unfortunately not collected as part of the SLIC Study, we included this in the limitations in the ‘Discussion’ section on lines 557-560 in the revised manuscript.

3) *Should the number of siblings or possibility to commute with friends/alone or proximity to public transport (tube station/bus stop) be taken into account in the analyses as possible confounders, i.e. should they be mentioned in the discussion as missing? Did you have such information available/was it asked?*

Similar to the information on parents’ and schools’ attitudes, this information was sadly unavailable for the SLIC sample. Hence, we also included this in the limitations in the ‘Discussion’ section on lines 557-560 in the revised manuscript.

4) *One minor detail in line 326 in page 15. I was wondering whether "or" would be better than "and" (...overweight OR obesity...)*

We agree with the reviewer that ‘or’ is better here, and have adjusted this accordingly on line 341 of the revised manuscript.
